# Western diet augments metabolic and arterial dysfunction in a sex-specific manner in outbred, genetically diverse mice

**DOI:** 10.3389/fnut.2022.1090023

**Published:** 2023-01-06

**Authors:** Xiangyu Zheng, Zhuoxin Li, Jennifer Berg Sen, Luaye Samarah, Christina S. Deacon, Joseph Bernardo, Daniel R. Machin

**Affiliations:** Department of Nutrition and Integrative Physiology, Florida State University, Tallahassee, FL, United States

**Keywords:** endothelium, fat, sugar, arterial function, blood pressure, aorta, metabolic function

## Abstract

Western diet (WD), characterized by excess saturated fat and sugar intake, is a major contributor to obesity and metabolic and arterial dysfunction in humans. However, these phenotypes are not consistently observed in traditional inbred, genetically identical mice. Therefore, we sought to determine the effects of WD on visceral adiposity and metabolic/arterial function in UM-HET3 mice, an outbred, genetically diverse strain of mice. Male and female UM-HET3 mice underwent normal chow (NC) or WD for 12 weeks. Body mass and visceral adiposity were higher in WD compared to NC (*P* < 0.05). Female WD mice had greater visceral adiposity than male WD mice (*P* < 0.05). The results of glucose and insulin tolerance tests demonstrated that metabolic function was lower in WD compared to NC mice (*P* < 0.05). Metabolic dysfunction in WD as was driven by male mice, as metabolic function in female WD mice was unchanged (*P* > 0.05). Systolic blood pressure (BP) and aortic stiffness were increased in WD after 2 weeks compared to baseline and continued to increase through week 12 (*P* < 0.05). Systolic BP and aortic stiffness were higher from weeks 2-12 in WD compared to NC (*P* < 0.05). Aortic collagen content was higher in WD compared to NC (*P* < 0.05). Carotid artery endothelium-dependent dilation was lower in WD compared to NC (*P* < 0.05). These data suggest sex-related differences in visceral adiposity and metabolic dysfunction in response to WD. Despite this, arterial dysfunction was similar in male and female WD mice, indicating this model may provide unique translational insight into similar sex-related observations in humans that consume WD.

## Introduction

Cardiovascular disease (CVD) is the leading cause of morbidity and mortality in industrialized societies and advanced age is the major risk factor for the development of CVD (1). In industrialized societies, advanced age is accompanied by visceral obesity and metabolic dysfunction (e.g., insulin resistance), as well as arterial dysfunction (i.e., increased systolic blood pressure [BP], stiffening of the large elastic arteries, and impaired endothelium-dependent dilation [EDD]) (2, 3). While visceral obesity, metabolic dysfunction, and arterial dysfunction are each independent risk factors for CVD (3, 4), CVD risk becomes even greater in individuals with combined risk factors (i.e., metabolic syndrome) (5). This becomes increasingly important in an aged society, as advancing age also increases the prevalence of metabolic syndrome (6).

Lifestyle factors, such as diet, are the most important modifiable risk factors for metabolic syndrome and subsequent CVD (7). Thus, consumption of a Western diet (WD) that consists of excess saturated fat and sugar intake (7), is reported to be a primary driver for metabolic syndrome and subsequent CVD risk in individuals living in the industrialized societies (8). This is evident as pre-industrialized societies shift toward industrialization, which increases the incidence of type-2 diabetes and CVD (9, 10). To determine the mechanisms by which a WD might elevate CVD risk, preclinical rodent models have been used to induce obesity, metabolic dysfunction, and arterial dysfunction. However, the vast majority of these studies use inbred, genetically identical mice, such as the C57BL/6 strain that have strain specific phenotypes that may limit translation to humans. For example, in response to WD, C57BL/6 mice display sex-differences in body mass gain (11–13) and/or a lack of elevation in systolic BP (14–19), which conflicts with observations in humans (20–22).

In recent years, outbred, genetically diverse mice, such as the UM-HET3 strain, have become popular in translational aging research, as they are hypothesized to better phenocopy the genetic diversity in humans (23), while displaying lower phenotypic variation than genetically identical inbred mice (24). However, very few studies have used outbred rodent models to study the effect of WD consumption. In outbred Sprague Dawley rats, studies show a large disparity in body mass gain to diet induced obesity (25–27), indicating a lack of consistency of this rat model. To the best of our knowledge, there is only one study that has used an outbred mouse model of genetic diversity with WD and showed that WD consumption induced a wide range of diabetes-related phenotypes (11). On the other side, the National Institute on Aging (NIA) Interventions Testing Program (ITP) and Study of Longitudinal Aging in Mice (SLAM) are longitudinal studies that target aging in UM-HET3 mice to establish normative aging values and identify potential therapeutics that extend lifespan (28–30). While the ITP and SLAM studies have strong translational importance, it is important to note that mice in these studies consume a standard normal chow (NC) diet. Thus, the translational application of these findings to industrialized societies in which many individuals consume a WD is unclear. To the best of our knowledge, there are no short- or long-term studies that have examined adiposity, metabolic function, and arterial function in UM-HET3 mice in response to a WD. Prior to performing a longitudinal study to assess the effects of WD on aging in UM-HET3 mice, it is imperative to first determine if the short-term adaptations to WD in young UM-HET3 mice phenocopy adaptations in humans to similar conditions. Therefore, the purpose of this study was to determine the effects of 12 weeks of WD consumption on adiposity, metabolic function, and arterial function in young UM-HET3 mice.

## Materials and methods

### Ethical approval

All animal procedures conform to the *Guide to the Care and Use of Laboratory Animals: Eighth Edition* (31) and were approved by the Florida State University Animal Care and Use Committee.

### Animals

Male and female UM-HET3 mice used in this study were first generation offspring of CByB6F1/J male and C3D2F1/J female parents. CByB6F1/J and C3D2F1/J parents were obtained from The Jackson Laboratory, as described previously (32). All mice were housed in standard mouse cages under a 12:12 light:dark cycle in a temperature-controlled environment. At 3 months old, mice were randomized into NC (LabDiet No. 5001; protein: 28.5%, carbohydrate: 58.0%, fat 13.5% by kcal; 1.1% NaCl) or WD (LabDiet No. 5TJN; protein: 15.8%, carbohydrate: 45.1%, fat 39.1% by kcal; 0.4% NaCl) groups for 12 weeks (33). The fat content in WD used in this study is comparable to typical WD used in most preclinical studies (34). Food and water were supplied *ad libitum* in group-housed cages. Body mass and blood glucose were measured in the non-fasting condition on the day of euthanasia. Mice were anesthetized with isoflurane (3%) in room air at 100 ml/min flow rate and euthanized via cardiac puncture.

### Blood collection and analyses

Blood was collected in the non-fasting condition via cardiac puncture and immediately centrifuged at 4°C for 20 min at 3,000 g. Plasma was aliquoted and stored at −80°C freezer. Plasma insulin was quantified using mouse insulin ELISA kit (Cat# 9008; Crystal Chem, Elk Grove Village, IL, USA).

### Metabolic testing

Metabolic function was determined using glucose- and insulin-tolerance tests (GTT, ITT) at baseline, after 6 weeks and 12 weeks of dietary intervention. Briefly, mice were fasted for 4 h in the morning. Baseline blood glucose was measured using a glucometer (Clarity BG1000; Clarity Diagnostics, Boca Raton, FL, USA) in blood collected via a tail cut. Following baseline measurements, mice were injected intraperitoneally with glucose (2 g/kg body mass) or insulin (1 U/kg body mass). Blood glucose was measured at 15, 30, 45, 60, 90, and 120 min after injection. HOMA-IR, a measurement of insulin sensitivity (35), was calculated using the equation: glucose × insulin ÷ 405.

### Systolic blood pressure

Arterial systolic BP was determined *in vivo* in conscious mice using the tail-cuff method (MC4000 BP analysis system; Hatteras Instrument, Cary, NC, USA), as described previously (36), at baseline and after 2, 4, 6, 8, 10, and 12 weeks of dietary intervention. This method has been validated vs. arterial catheter BP (37). Briefly, mice underwent a 5 consecutive days of tail cuff BP measurement that was conducted in a quiet and warm (∼23°C) environment at the same time of the day (38). During each trial, mice were restrained on a heated platform (40°C). Each trial consisted of 5 preliminary measurements that were followed by 10 experimental measurements. Measurements with aberrant movement/behavior or poor signal were excluded and remaining values were used to calculate mean values for each mouse.

### Aortic stiffness

Aortic stiffness was assessed by aortic pulse wave velocity (PWV) measurement *in vivo*, as described previously (39), at baseline and after 2, 4, 6, 8, 10, and 12 weeks of dietary intervention. Briefly, mice were anesthetized with isoflurane (3%) in room air at 100 ml/min flow rate and placed in the supine position on a heated platform (37°C). Blood velocity waveforms at the transverse aortic arch and at the abdominal aorta were obtained simultaneously with 2, 20-MHz Doppler probes (Indus Instruments, Webster, TX, USA) and recorded using PowerLab 16/35 with LabChart 8 software (AD Instruments Inc., Colorado Springs, CO, USA) at a sampling rate of 100 k/sec. After blood velocity waveforms were collected, a precise measurement of the traveled distance between the Doppler probes was recorded using a scientific caliper. The transit time between Doppler sites was determined using the foot-to-foot method with LabChart Lightning 1.8 software (AD Instruments Inc., Colorado Springs, CO, USA). Aortic PWV was calculated as the traveled distance divided by the transit time.

### *Ex vivo* arterial function

To assess EDD, carotid arteries were excised, cleared of surrounding tissue, cannulated in the stage of a pressure myograph (DMT Inc., Hinnerup, Denmark), and perfused with physiological salt solution that contained 145.0 mM NaCl, 4.7 mM KCl, 2.0 mM CaCl_2_, 1.17 mM MgSO_4_, 1.2 mM NaH_2_PO_4_, 5.0 mM glucose, 2.0 mM pyruvate, 0.02 mM EDTA, 3.0 mM MOPS buffer, and 0.5% BSA (pH 7.4 at 37°C). Carotid arteries were pressurized to an intraluminal pressure of 68 cm H_2_O. Arteries were submaximally pre-constricted with 2 μM phenylephrine and EDD was measured in response to the cumulative addition of acetylcholine (1×10^–10^ to 1×10^–4^ M) in the absence or presence of the nitric oxide (NO) synthase inhibitor, L-NAME (0.1 mM, 30 min), as described previously (40). Endothelium-independent dilation (EID) was assessed in response to the cumulative addition of sodium nitroprusside (1×10^–10^ to 1×10^–4^ M). Following *ex vivo* measurements, carotid arteries were incubated in Ca^2+^-free physiological salt solution for 1 hour to determine their maximal diameter. Luminal diameters were measured by Vasotracker software 2 minutes after each addition of acetylcholine or sodium nitrorpsuside (41). All *ex vivo* arterial function data are presented as percent of maximal dilation after pre-constriction with phenylephrine. Arteries failing to achieve ≥ 15% pre-constriction were excluded.

### Aortic histology

After sacrifice, a 2-3 mm aortic ring with perivascular tissue intact was excised from the thoracic aorta and embedded in Optimal Cutting Temperature medium, as described previously (39). Aortic rings were sliced into 8-micron sections. Each slide contained 2 to 3 aortic sections, which were averaged. For measures of medial cross-sectional area (CSA) the lumen border and the outer medial border were traced in ImageJ and internal areas were measured. These areas were used to calculate medial CSA and were calculated as the outer medial border area minus the lumen area. Collagen was quantified by Masson’s trichrome staining (MilliporeSigma, Burlington, MA, USA) as percentage of the selected area, as described previously (42). Blue channel images from an RGB stack were used for densitometric quantification of collagen content with ImageJ. Elastin was quantified by Verhoff-Van Gieson staining (Abcam, Cambridge, UK), as described previously (39). An 8-bit grayscale was used for densitometric quantification of elastin content with ImageJ. Collagen and elastin content were normalized to NC.

### Statistical analysis

A 4-way mixed model ANOVA was used to evaluate the effect of Sex, Group, Week, Time/Concentration on GTT/ITT and *ex vivo* arterial function responses. A 3-way mixed model ANOVA was used to evaluate the effect of Sex, Group, and Week on systolic BP, aortic PWV, GTT/ITT AUC, and *ex vivo* arterial function responses at each drug concentration. To analyze the remaining data, a 2-way ANOVA or 2-way mixed model ANOVA was used to evaluate the effect of Sex, Group, or Time/Concentration on all the variables. Sidak *post-hoc* test was used to further identify values that were significantly different. Bivariate correlations were determined between selected variables. Statistical significance was set at *P* < 0.05 for all analyses. Statistic tests were performed with SPSS software version 26.0 (IBM, Armonk, NY, USA). Data are presented as mean ± SEM.

## Results

### Dietary intake

Dietary intake is presented in Table 1. There were Sex-related differences in energy intake (*P* < 0.05) but no differences in energy intake were present between groups (*P* > 0.05). There were also Group- and Sex-related differences in carbohydrate, fat, and protein intake (*P* < 0.05). No interaction effects were observed (*P* > 0.05).

**TABLE 1:** Dietary intake.

	Normal chow		Western diet				
	Male	Female	Male	Female	Group	Sex	Interaction
Sample size, n	6	6	6	6	−	—	—
Energy intake, kcal/d	17.2 ± 0.4	15.9 ± 0.5	17.1 ± 1.2	14.7 ± 0.1	0.329	0.010	0.392
Carbohydrate intake, kcal/d	10 ± 0.2	9.2 ± 0.3	7.7 ± 0.5	6.6 ± 0	<0.001	0.008	0.643
Fat intake, kcal/d	2.3 ± 0.1	2.1 ± 0.1	6.7 ± 0.5	5.7 ± 0	<0.001	0.022	0.105
Protein intake, kcal/d	4.9 ± 0.1	4.5 ± 0.1	2.7 ± 0.2	2.3 ± 0	<0.001	0.008	0.846

Values are means ± SEM. Data were analyzed using 2-way ANOVA. Sidak *post-hoc* test was used to identify group differences.

### Animal characteristics

**FIGURE 1:**
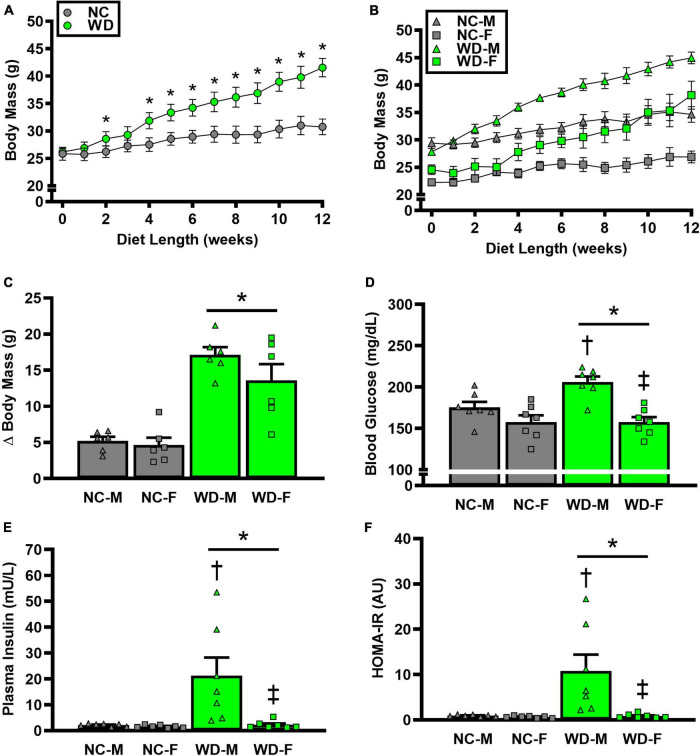
Comparisons in normal chow (NC) and Western (WD) diet-treated male (M) and female (F) mice. Data were analyzed using 2-way and 3-way mixed model ANOVA and 2-way ANOVA. Sidak *post-hoc* test was used to identify differences in body mass between groups **(A)** and sexes **(B)**, as well as differences in Δ body mass **(C)**, non-fasting blood glucose **(D)**, non-fasting plasma insulin **(E)**, and HOMA-IR **(F)** between groups/sexes. *P < 0.05 vs. NC. ^†^*P*, 0.05 vs. NC within sex. ^‡^*P* < 0.05 vs. male within group. Data are individual values and means ± SEM.

We observed a significant main effect of Group and Time, as well as a significant interaction effect of Group X Week on body mass and change in body mass (Figure 1; *P* < 0.05 for all). At baseline (i.e., week 0), body mass was similar between NC and WD (Figure 1A; *P* > 0.05). Over the 12-week dietary intervention, body mass was increased from baseline in WD mice at week 2 and continued to increase through week 12 (*P* < 0.05). With the exception of week 3, body mass was greater in WD compared to NC mice in weeks 2-12 (*P* < 0.05). Body mass also increased in NC mice at week 6 and weeks 10-12 compared to baseline (*P* < 0.05). While we did observe a significant main effect of Sex (*P* < 0.05), there was no interaction effect of Sex with body mass (Figure 1B; *P* > 0.05) or change in body mass (Figure 1C; P > 0.05). However, the change in body mass over the 12-week intervention was ∼3-fold greater in WD compared to NC mice (Figure 1C; *P* < 0.05). Visceral adipose tissue mass expressed as an absolute mass, as well as normalized to body mass or tibia length were higher in WD compared to NC mice (Table 2; *P* < 0.05). We also observed sex-related differences in visceral adiposity with female WD mice having greater absolute and relative visceral adiposity mass compared to male WD mice (*P* < 0.05).

**TABLE 2:** Animal characteristics.

	Normal chow		Western diet				
	Male	Female	Male	Female	Group	Sex	Interaction
Sample size, n	6	6	6	6	−	−	—
Age, mo	6.7 ± 0.3	6.9 ± 0.0	6.5 ± 0.2	6.9 ± 0.0	0.674	<0.001	0.104
Body mass, g	34.7 ± 1.5	26.7 ± 1.1	45.2 ± 2.2	38.3 ± 2.8	<0.001	0.002	0.802
Tibia length, mm	18.6 ± 0.2	20.0 ± 0.5	19.1 ± 0.3	19.3 ± 0.3	0.876	0.048	0.145
Heart, mg	169 ± 9	139 ± 5	188 ± 12	139 ± 6	0.295	<0.001	0.253
Heart/Body mass, mg/g	4.9 ± 0.2	5.2 ± 0.2	4.2 ± 0.3	3.7 ± 0.3	0.001	0.828	0.163
Heart/Tibia length, mg/mm	9.1 ± 0.5	7.0 ± 0.2	9.8 ± 0.5	7.2 ± 0.3	0.258	<0.001	0.495
Liver, mg	1739 ± 80	1400 ± 68	2233 ± 188	1509 ± 121	0.024	<0.001	0.135
Liver/Body mass, mg/g	50.4 ± 2.2	52.5 ± 2.1	49.1 ± 2.4	40.1 ± 3.3*^†^	0.014	0.191	0.042
Liver/Tibia length, mg/mm	93.4 ± 3.8	70.2 ± 2.9	116.6 ± 9.0	77.8 ± 5.3	0.014	<0.001	0.189
Spleen, mg	78 ± 8	90 ± 3	86 ± 8	94 ± 7	0.365	0.157	0.742
Spleen/Body mass, mg/g	2.3 ± 0.3	3.4 ± 0.2	1.9 ± 0.1	2.5 ± 0.2	0.003	<0.001	0.181
Spleen/Tibia length, mg/mm	4.2 ± 0.4	4.5 ± 0.2	4.5 ± 0.4	4.8 ± 0.3	0.321	0.311	0.972
Visceral adipose tissue, mg	431 ± 78	255 ± 108	901 ± 101*	1403 ± 251*^†^	<0.001	0.295	0.036
Visceral adipose tissue/Body mass, mg/g	12.1 ± 1.8	8.8 ± 3.5	19.8 ± 1.6	35.3 ± 5.0*^†^	<0.001	0.075	0.009
Visceral adipose tissue/Tibia length, mg/mm	23.0 ± 4.0	12.1 ± 4.9	47.1 ± 5.0*	73.0 ± 13.7*^†^	<0.001	0.356	0.031
Quadricep, mg	240 ± 6	194 ± 18	216 ± 15	211 ± 6	0.782	0.049	0.104
Quadricep/Body mass, mg/g	7.0 ± 0.4	7.4 ± 0.9	4.8 ± 0.4	5.6 ± 0.4	0.002	0.298	0.701
Quadricep/Tibia length, mg/mm	12.9 ± 0.5	9.8 ± 1.0	11.3 ± 0.9	10.9 ± 0.4	0.749	0.028	0.083
Gastrocnemius, mg	164 ± 4	135 ± 9	166 ± 12	115 ± 7	0.269	<0.001	0.195
Gastrocnemius/Body mass, mg/g	4.8 ± 0.2	5.1 ± 0.4	3.7 ± 0.3	3.1 ± 0.3	0.000	0.677	0.163
Gastrocnemius/Tibia length, mg/mm	8.8 ± 0.3	6.8 ± 0.5	8.6 ± 0.5	5.9 ± 0.4	0.239	<0.001	0.423
Soleus, mg	11 ± 1	8 ± 1	11 ± 1	10 ± 2	0.680	0.085	0.583
Soleus/Body mass, mg/g	0.3 ± 0.0	0.3 ± 0.0	0.2 ± 0.0	0.2 ± 0.0	0.025	0.844	0.715
Soleus/Tibia length, mg/mm	0.6 ± 0.0	0.4 ± 0.0	0.6 ± 0.1	0.5 ± 0.1	0.735	0.025	0.389
Plantaris, mg	22 ± 1	21 ± 1	24 ± 1	18 ± 2	0.664	0.014	0.065
Plantaris/Body mass, mg/g	0.6 ± 0.0	0.8 ± 0.1^†^	0.5 ± 0.0	0.5 ± 0.1*	<0.001	0.331	0.041
Plantaris/Tibia length, mg/mm	1.2 ± 0.0	1.1 ± 0.1	1.3 ± 0.0	0.9 ± 0.1	0.601	0.002	0.110
Kidney, mg	297 ± 13	215 ± 8	293 ± 22	201 ± 11	0.525	<0.001	0.745
Kidney/Body mass, mg/g	8.6 ± 0.5	8.1 ± 0.3	6.5 ± 0.5	5.4 ± 0.5	<0.001	0.081	0.531
Kidney/Tibia length, mg/mm	16 ± 0.7	10.8 ± 0.4	15.2 ± 0.9	10.4 ± 0.4	0.358	<0.001	0.835
Blood glucose, mg/dl	175 ± 7	158 ± 8	206 ± 7*	158 ± 6^†^	0.036	<0.001	0.033
Plasma insulin, mU/L	2.2 ± 0.2	1.6 ± 0.2	21.2 ± 8.3*	2.2 ± 0.6^†^	0.014	0.014	0.020
HOMA-IR, U	0.9 ± 0.1	0.6 ± 0.1	10.7 ± 4.3*	0.8 ± 0.2^†^	0.015	0.013	0.019

Values are means ± SEM. Data were analyzed using 2-way ANOVA. Sidak post-hoc test was used to identify group differences.

*P < 0.05 vs. normal chow diet within sex.

^†^*P* < 0.05 vs. male within group.

### Metabolic function

We observed a significant main effect of Group and Sex, as well as a significant interaction effect of Group X Sex with non-fasted blood glucose, plasma insulin, and HOMA-IR (Figure 1; *P* < 0.05 for all). Non-fasted blood glucose, plasma insulin, and HOMA-IR were also higher in WD compared to NC mice (Figures 1D-F; *P* < 0.05). These group differences were driven by male WD mice, as they had higher blood glucose, plasma insulin, and HOMA-IR compared to female WD mice (*P* < 0.05). There were no sex-related differences in these variables between male and female NC mice (*P* > 0.05).

**FIGURE 2:**
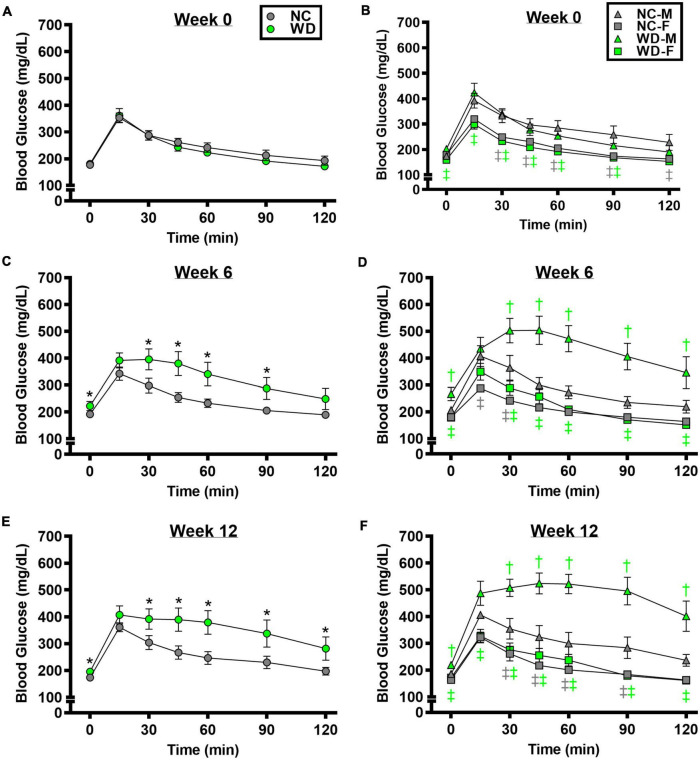
Comparisons in normal chow (NC) and Western (WD) diet-treated male (M) and female (F) mice. Data were analyzed using 3-way and 4-way mixed model ANOVA. Sidak *post-hoc* test was used to identify differences in blood glucose in response to glucose tolerance test between groups **(A)** and sexes **(B)** at week 0, week 6 **(C,D)**, and week 12 **(E,F)**. *P < 0.05 vs. NC. ^†^*P* < 0.05 vs. NC within sex. ^‡^*P* < 0.05 vs. male within group. Data are means ± SEM.

We observed a significant main effect of Group, Week, and Time, as well as a significant interaction effects of Group X Week and Group X Week X Time with GTT response (Figure 2; *P* < 0.05). At baseline, glucose tolerance was similar between groups (Figure 2A; *P* > 0.05). However, glucose tolerance was lower in WD compared to NC mice at weeks 6 and 12 (Figures 2C,E; *P* < 0.05). We also observed a significant main effect of Sex, as well as significant interaction effects of Sex X Group, Sex X Week, Sex X Group X Week X Time for GTT response (*P* < 0.05). At baseline (Figure 2B), week 6 (Figure 2D), and week 12 (Figure 2F) timepoints, glucose tolerance was lower in males compared to females (*P* < 0.05). Although female NC and WD mice had similar glucose tolerance at all timepoints (*P* > 0.05), only at baseline was glucose tolerance similar between male NC and WD mice (*P* > 0.05). However, at weeks 6 and 12, glucose tolerance was lower in male WD compared to male NC mice (*P* < 0.05).

**FIGURE 3:**
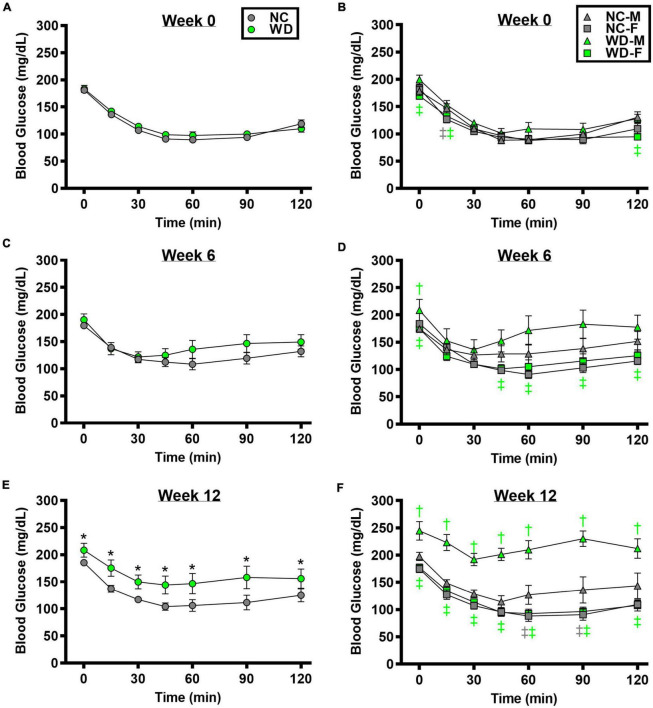
Comparisons in normal chow (NC) and Western (WD) diet-treated male (M) and female (F) mice. Data were analyzed using 3-way and 4-way mixed model ANOVA. Sidak *post-hoc* test was used to identify differences in blood glucose in response to insulin tolerance test between groups **(A)** and sexes **(B)** at week 0, week 6 **(C,D)**, and week 12 **(E,F)**. *P < 0.05 vs. NC. ^†^*P* < 0.05 vs. NC within sex. ^‡^*P* < 0.05 vs. male within group. Data are means ± SEM.

We observed a significant main effect of Group, Week, and Time, as well as a significant interaction effects of Group X Week and Group X Week X Time with ITT response (Figure 3; *P* < 0.05). At baseline and week 6, insulin tolerance was similar between groups (Figures 3A,C; *P* > 0.05), although insulin tolerance was lower in WD compared to NC mice at week 12 (Figure 3E; *P* < 0.05). We also observed a significant main effect of Sex, as well as significant interaction effects of Sex X Group, Sex X Week, and Sex X Group X Week X Time with ITT response (*P* < 0.05). At baseline (Figure 3B), week 6 (Figure 3D), and week 12 (Figure 3F) timepoints, insulin tolerance was lower in males compared to females (*P* < 0.05). Although female NC and WD mice had similar insulin tolerance at all timepoints (*P* > 0.05), only at baseline was insulin tolerance similar between male NC and WD mice (*P* > 0.05). However, at weeks 6 and 12, insulin tolerance was lower in male WD compared to male NC mice (*P* < 0.05).

**FIGURE 4:**
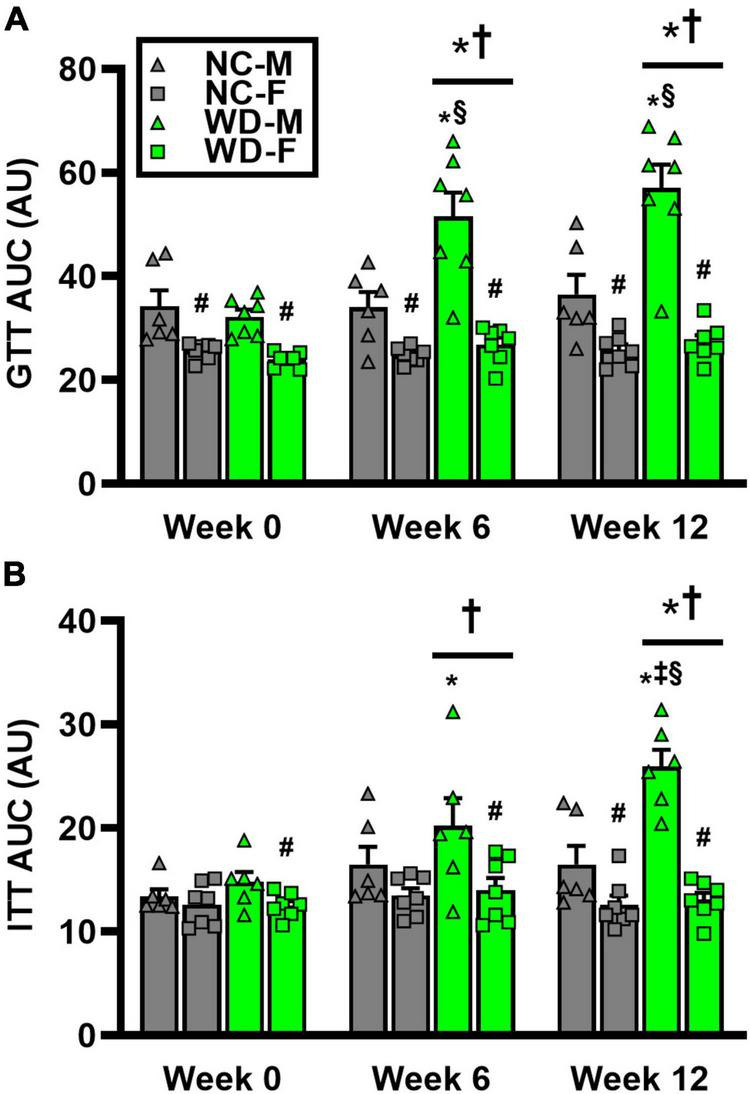
Comparisons in normal chow (NC) and Western (WD) diet-treated male (M) and female (F) mice. Data were analyzed using 2-way and 3-way mixed model ANOVA. Sidak *post-hoc* test was used to identify differences in glucose tolerance test (GTT) area under the curve (AUC) between groups/sexes **(A)**, as well as differences in insulin tolerance test (ITT) AUC between groups/sexes **(B)**. *P < 0.05 vs. NC. ^†^*P* < 0.05 vs. Week 0. ^‡^*P* < 0.05 vs. Week 6. ^§^*P* < 0.05 vs. NC within sex. ^#^*P* < 0.05 vs. male within group. Data are individual values and means ± SEM.

We observed a significant main effect of Group and Time, as well as a significant interaction effect with Group X Time with GTT area under the curve (AUC) (Figure 4; *P* < 0.05). At baseline, GTT AUC was similar between NC and WD (Figures 4A; *P* < 0.05). However, GTT AUC was higher in WD compared to NC at weeks 6 and 12 (*P* < 0.05). We also observed a significant main effect of Sex as well as significant interaction effects of Sex X Group, Sex X Time, and Sex X Group X Time with GTT AUC (*P* < 0.05). Within both diet groups, female mice had a lower GTT AUC compared to male mice at all weeks (Figure 4A; *P* < 0.05). Additionally, we observed elevations in GTT AUC in WD male mice compared to NC males at weeks 6 and 12 (*P* < 0.05). We observed a significant main effect of Group and Time, as well as a significant interaction effect with Group X Time with ITT AUC (Figure 4; *P* < 0.05). At baseline and week 6, ITT AUC was similar between NC and WD (Figure 4B; *P* < 0.05). However, ITT AUC was higher in WD compared to NC at week 12 (*P* < 0.05). Also, we observed a significant main effect of Sex as well as significant interaction effects of Sex X Group, Sex X Time, and Sex X Group X Time with ITT AUC (*P* < 0.05). Within both diet groups, female mice had a lower ITT AUC compared to male mice at most weeks (Figure 4B; *P* < 0.05). Additionally, we observed elevations in ITT AUC in WD male mice compared to NC males at weeks 6 and 12 (*P* < 0.05).

### Systolic blood pressure

**FIGURE 5:**
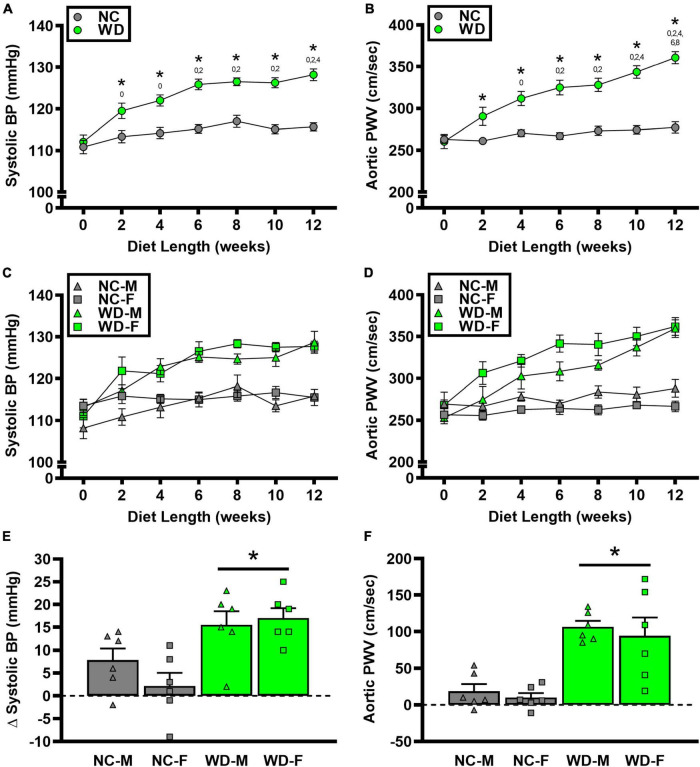
Comparisons in normal chow (NC) and Western (WD) diet-treated male (M) and female (F) mice. Data were analyzed using 2-way and 3-way mixed model ANOVA and 2-way ANOVA. Sidak *post-hoc* test was used to identify group differences in systolic blood pressure (BP) **(A)** and aortic pulse wave velocity (PWV) **(B)**, sex-differences in systolic BP **(C)** and aortic PWV **(D)**, as differences in Δ systolic BP **(E)** and Δ aortic PWV **(F)** between groups/sexes. *P < 0.05 vs. NC. 0, 2, 4, 6, 8, 10 *P* < 0.05 vs. the corresponding week, respectively. Data are individual values and means ± SEM.

We observed a significant main effect of Group and Week, as well as a significant interaction effect of Group X Week with systolic BP and change in systolic BP (Figure 5; *P* < 0.05 for all). At baseline, systolic BP was similar between NC and WD (Figure 5A; *P* > 0.05). Over the 12-week dietary intervention, systolic BP increased from baseline in WD mice at week 2 and continued to increase through week 12 (*P* < 0.05). Systolic BP was greater in WD compared to NC mice in weeks 2-12 (*P* < 0.05). We did not observe any sex-related differences in systolic BP (Figure 5C; *P* > 0.05) or change in systolic BP (Figure 5E; *P* > 0.05). The change in systolic BP over the 12-week intervention was ∼3-fold greater in WD compared to NC mice (Figure 5E; *P* < 0.05).

### Aortic structure and function

**FIGURE 6:**
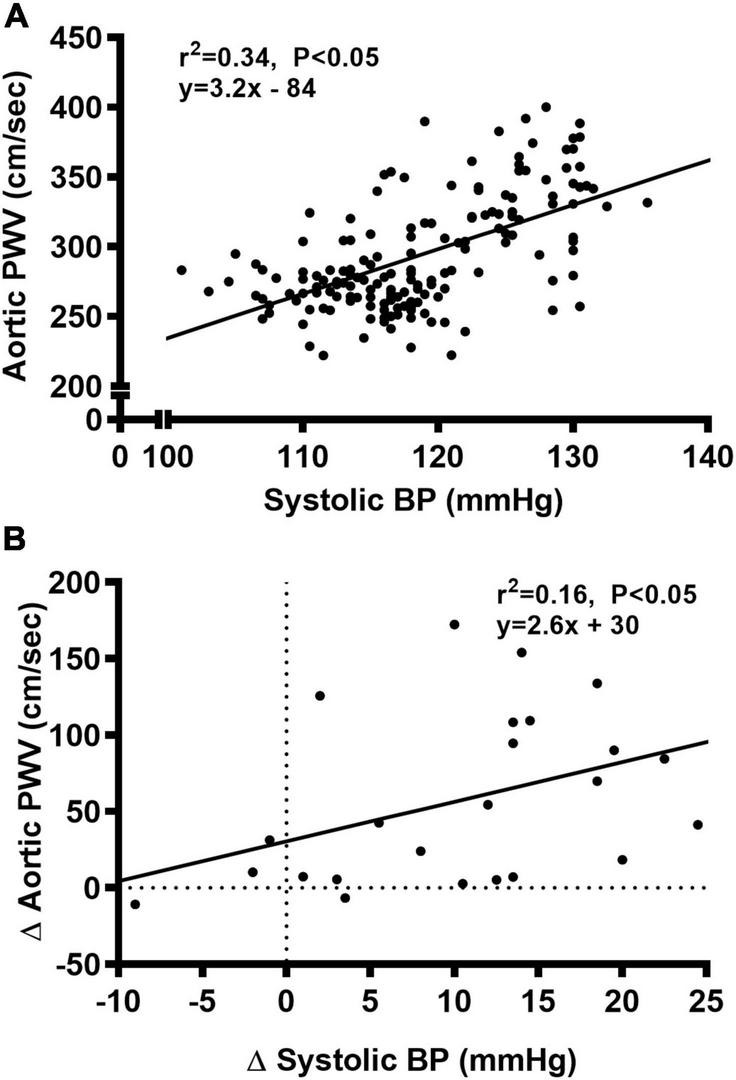
Bivariate correlational analysis was used to determine the relationship between aortic pulse wave velocity (PWV) and systolic blood pressure (BP) **(A)**, as well as the relationship between Δ in systolic BP and aortic PWV **(B)** in normal chow and Western diet-treated mice. Data are individual values.

**FIGURE 7:**
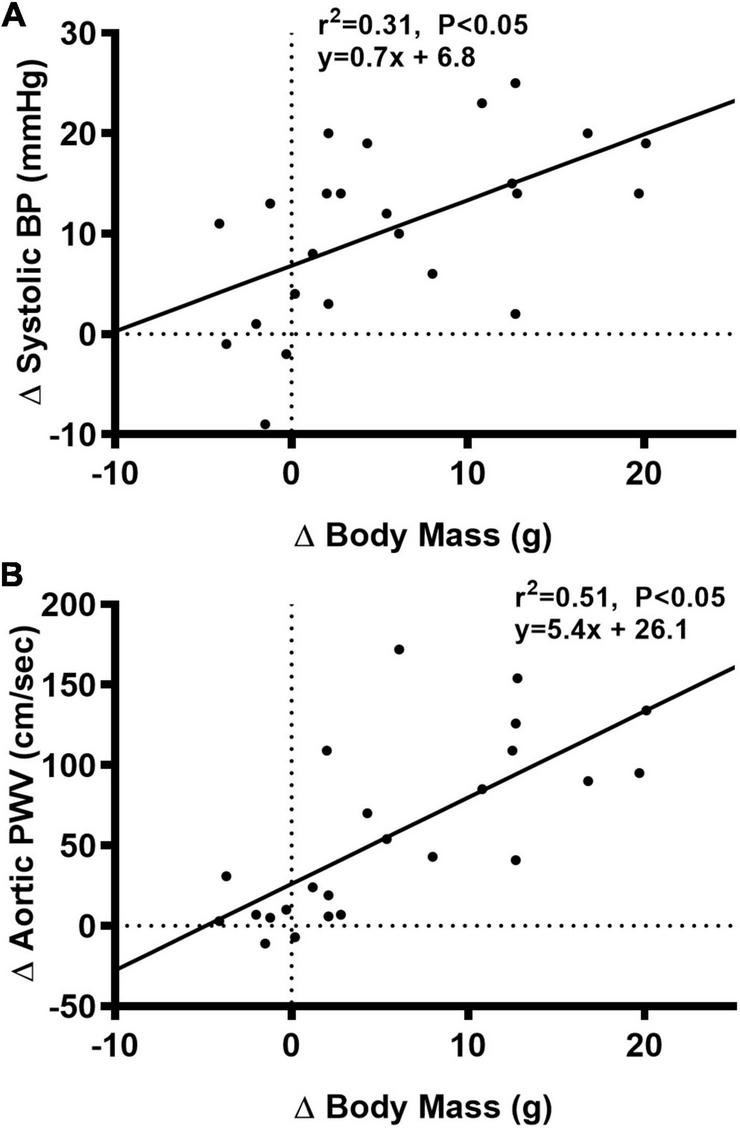
Bivariate correlational analysis was used to determine the relationship between Δ in systolic blood pressure (BP) **(A)** or aortic pulse wave velocity (PWV) **(B)** with Δ in body mass in normal chow and Western diet-treated mice. Data are individual values.

We observed a significant main effect of Group and Week, as well as a significant interaction effect of Group X Week with aortic PWV and change in aortic PWV (Figure 5; *P* < 0.05 for all). At baseline, aortic PWV was similar between NC and WD (Figure 5B; *P* > 0.05). Over the 12-week dietary intervention, aortic PWV increased from baseline in WD mice at week 4 and continued to increase through week 12 (*P* < 0.05). Aortic PWV was greater in WD compared to NC mice in weeks 2-12 (*P* < 0.05). We observed a significant interaction effect of Sex X Group with aortic PWV (*P* < 0.05), indicating female WD mice had greater aortic PWV than male WD mice (Figure 5D; *P* < 0.05). There was no sex-related difference in change in aortic PWV (Figure 5F; *P* > 0.05). The change in aortic PWV over the 12-week intervention was ∼7-fold greater in WD compared to NC mice (Figure 5F; *P* < 0.05). There was a strong correlation between aortic stiffness and systolic BP (Figure 6A, *r*^2^ = 0.34, *P* < 0.05), as well as a moderate correlation between change in aortic stiffness and systolic BP (Figure 6B, *r*^2^ = 0.16, *P* < 0.05). We also observed strong correlations between change in body mass and change in systolic BP (Figure 7A, *r*^2^ = 0.31, *P* < 0.05) or aortic stiffness (Figure 7B, *r*^2^ = 0.51, *P* < 0.05).

**FIGURE 8:**
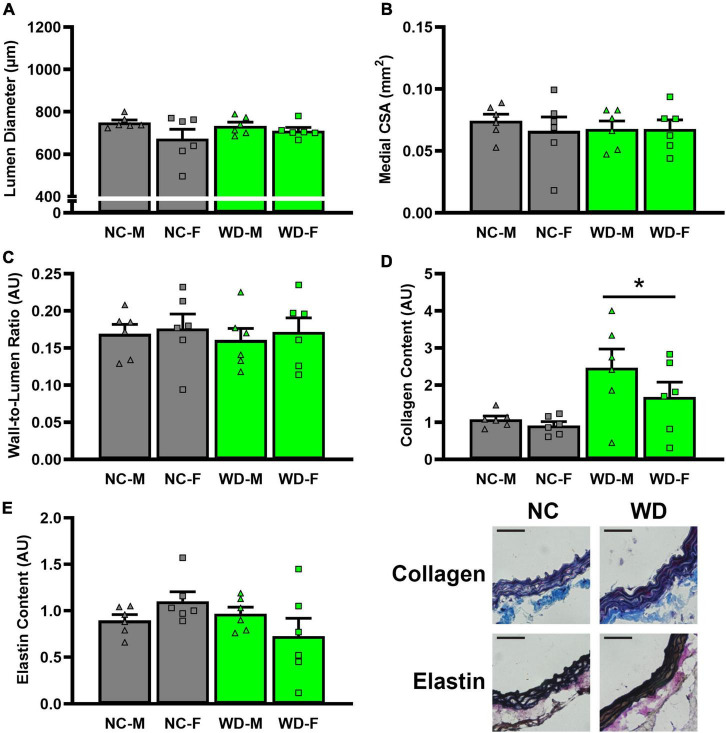
Comparisons in normal chow (NC) and Western (WD) diet-treated mice. Data were analyzed using 3-way ANOVA. Sidak *post-hoc* test was used to identify groups/sex differences in aortic lumen diameter **(A)**, medial cross-sectional area (CSA) **(B)**, wall-to-lumen area **(C)**, collagen **(D)**, and elastin **(E)** content. Figures are accompanied by representative images of collagen and elastin staining. Black scale bars are equal to 500 μm. *P < 0.05 vs. NC. Data are individual values and means ± SEM.

In histological sections of thoracic aortas, there was no difference in lumen diameter (Figure 8A, *P* > 0.05), medial CSA (Figure 8B, *P* > 0.05), or medial wall-to-lumen ratio (Figure 8C, *P* > 0.05) between groups. Collagen content was elevated in WD compared to NC (Figure 8D, *P* < 0.05), but there were no differences in elastin content (Figure 8E, *P* > 0.05). There were no sex-related differences in histological analyses (*P* > 0.05).

### *Ex vivo* arterial function

**FIGURE 9:**
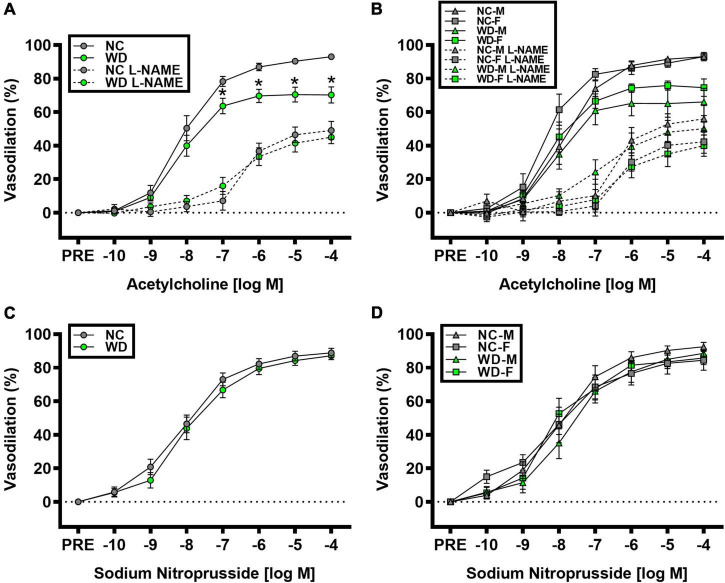
Comparisons in normal chow (NC) and Western (WD) diet-treated male (M) and female (F) mice. Data were analyzed using 2-way and 3-way mixed model ANOVA. Sidak *post-hoc* test was used to identify differences in carotid artery vasodilation to acetylcholine in the presence and absence of L-NAME between groups **(A)** and sexes **(B)**, as well as to identify differences in carotid artery vasodilation to sodium nitroprusside between groups **(C)** and sexes **(D)**. *P < 0.05 vs. NC. Data are means ± SEM.

**FIGURE 10:**
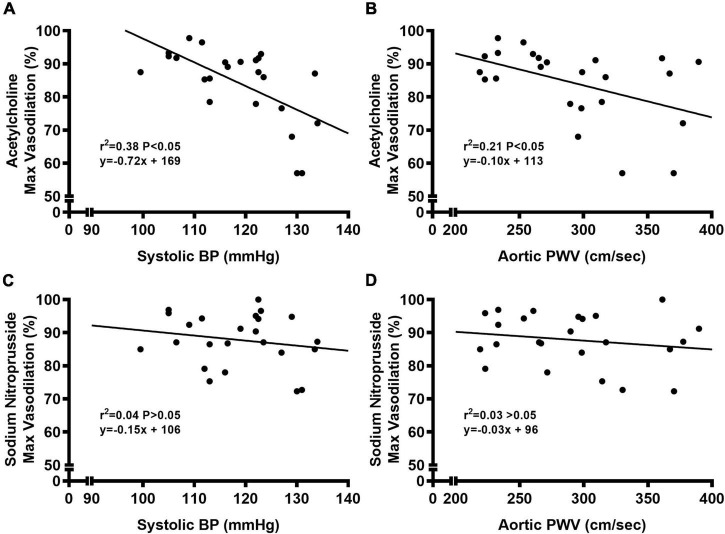
Bivariate correlational analysis was used to determine relations between maximal vasodilation to acetylcholine with systolic blood pressure (BP) **(A)** or aortic pulse wave velocity (PWV) **(B)**, as well as relations between maximal vasodilation to sodium nitroprusside with systolic blood pressure (BP) **(C)** or aortic pulse wave velocity (PWV) **(D)** in normal chow and Western diet-treated mice. Data are individual values.

We observed a significant main effect of Group and Concentration, as well as a significant interaction effect of Group X Concentration with carotid artery vasodilation to acetylcholine (Figure 9; *P* < 0.05 for all). Carotid artery EDD was higher in NC compared to WD mice (Figure 9A; *P* < 0.05). In the presence of L-NAME, EDD was lower in both NC and WD (*P* < 0.05 vs. acetylcholine), but similar between groups (*P* > 0.05). There were no sex-related differences in EDD (Figure 9B; *P* > 0.05). We observed a significant main effect of Concentration with carotid artery vasodilation to sodium nitroprusside (Figure 9C; *P* < 0.05), but there were no group differences in EID (*P* > 0.05). There were no sex-related differences in EID (Figure 9D; *P* > 0.05). We observed no differences in carotid artery maximal luminal diameter (NC-Male: 436 ± 10; NC-Female: 421 ± 10; WD-Male: 444 ± 5; WD-Female: 438 ± 9 μm; *P* > 0.05), wall thickness (NC-Male: 51 ± 3; NC-Female: 51 ± 3; WD-Male: 55 ± 3; WD-Female: 47 ± 4 μm; *P* > 0.05), or phenylephrine-induced preconstriction (NC-Male: 24.5 ± 3.2; NC-Female: 22.0 ± 3.3; WD-Male: 25.7 ± 3.6; WD-Female: 23.6 ± 2.2 % preconstriction; *P* > 0.05). There were strong, inverse correlations between maximal vasodilation to acetylcholine and systolic BP (Figure 10A, *P* < 0.05) or aortic stiffness (Figure 10B, *P* < 0.05). However, no relationship between systolic BP (Figure 10C, *P* > 0.05) or aortic stiffness (Figure 10D, *P* > 0.05) and maximal vasodilation to sodium nitroprusside was present.

## Discussion

In the present study, 12 weeks of WD resulted in increased visceral adiposity, metabolic dysfunction, and arterial dysfunction in outbred, genetically diverse young mice. Interestingly, visceral adiposity was observed in both male and female WD mice. However, metabolic dysfunction (i.e., impaired GTT and ITT responses and elevated HOMA-IR) was only observed in male WD mice. Unlike metabolic dysfunction, arterial dysfunction was present in both male and female WD mice, which was indicated by augmented systolic BP and aortic PWV and impaired EDD. WD-induced arterial dysfunction was also accompanied by structural adaptations within the aorta that resulted in augmented aortic collagen content. Taken together, these findings provide evidence that WD-induced visceral adiposity and arterial dysfunction occur in male and female UM-HET3 mice, despite preserved metabolic function in WD-fed female mice.

### Sex-related differences in metabolic response to Western diet

Body mass and visceral adiposity were higher in male and female WD mice, compared to their NC counterparts. Although the change in body mass in response to WD was similar between males and females, visceral adiposity was ∼56% heavier in female WD mice compared to male WD mice. We observed no sex-related differences in visceral adiposity between male and female NC mice. Thus, sex-related differences in visceral adiposity between male and female WD mice indicate that WD exerts a greater obesogenic effect on female mice. As a group, metabolic dysfunction was worse in WD compared to NC mice. Interestingly, this was entirely driven by worsened metabolic dysfunction in male WD mice. Indeed, compared to NC mice and female WD mice, male WD mice had a higher non-fasted blood glucose, plasma insulin concentrations, and HOMA-IR. Moreover, male WD mice had worse glucose and insulin tolerance in response to GTTs and ITTs, respectively, compared to NC mice and female WD mice. Indeed, GTT and ITT responses were similar at all timepoints (i.e., 0-, 6-, and 12-weeks) between NC and WD female mice. It has been previously reported that female mice have greater insulin sensitivity in diet-induced obesity models (13, 43), and this has also been shown in women (44, 45). Thus, female mice may have a greater ability to store adipose tissue, which is critical to maintain insulin sensitivity (43, 46), and might explain why metabolic function was preserved in female WD mice. Although this appears to be a beneficial feature in female mice, we still observed similar or worse arterial dysfunction in female WD mice, compared to male WD mice. Thus, further study in this area is warranted.

### Western diet augments systolic blood pressure and aortic stiffness

Despite sex-related differences in metabolic function in WD mice, we observed a similar time course of elevation in systolic BP between male and female across the 12-week intervention. Indeed, 2 weeks after beginning WD, systolic BP was increased in male and female mice. Systolic BP continued to rise throughout the 12-week dietary intervention. These data are in contrast to other studies that have shown a minimal effect of WD on systolic BP in mice (14–19). However, it is important to note that these studies used genetically identical, inbred mouse strains, such as C57BL/6. Moreover, WD consumption in human is associated with elevated systolic BP (47–49). Also, there is direct evidence demonstrating experimental weight gain over a short period of time in humans increases systolic BP (20–22). Thus, the lack of a hypertensive effect of WD in inbred mice may be a strain-specific phenotype that may be avoided by using outbred, genetically diverse mice, which also increases the translatability of the current findings to humans. Thus, our findings in the present study that UM-HET3 mice more closely resemble systolic BP responses to WD and its association to weight gain in humans should be considered as a translational strength of this study and this mouse model.

Elevated systolic BP in WD mice was accompanied by augmented aortic stiffness. Although increases in systolic BP do not commonly occur this early after beginning WD in inbred strains, augmented aortic stiffening has been shown in response to WD (50–53). To the best of our knowledge, the present study is the first to examine systolic BP and aortic stiffening in response to WD in UM-HET3 mice. Early in the intervention, systolic BP and aortic stiffness tended to increase at a similar rate across the 12-week intervention. However, in the latter weeks of the intervention aortic stiffness appeared to continue increases at the same rate, while increases in systolic BP tended to slow down.

Precise determination of whether elevations in systolic BP augment aortic stiffness or vice versa with WD is beyond the scope of this study. However, some mechanistic insight may be achieved by examining the time course of changes in these measurements. Changes in systolic BP and aortic stiffness were also accompanied by significant increases in body mass. While aortic stiffening has been shown to precede elevations in systolic BP in WD-fed C57BL/6 mice (54), these changes were shown to be dependent on adiposity, as reductions in adiposity lead to a decrease in systolic BP and aortic stiffness. We observed a strong relationship between the body mass gain and changes in systolic BP. In addition to augmented systolic BP and aortic stiffness, aortic collagen content was also augmented in WD mice. Increased arterial collagen content is a hallmark of arterial aging and is thought to be due to age-related changes to aortic structure and function (55). Thus, it is possible that the increase in collagen content represents arterial remodeling in response to WD consumption, and this remodeling is responsible for the continued increase in aortic stiffness and systolic BP throughout the intervention (56, 57). The direct mechanism responsible for augmented aortic stiffness cannot be determined from this study. However, modest weight gain has been associated with increased arterial stiffness in nonobese men but not nonobese women (58), although total and visceral adiposity have been shown to have a greater association with arterial stiffness in obese women than obese men (59). To the best of our knowledge, only one study measured systolic BP and aortic stiffness across the lifespan of WD-fed C57BL/6 mice and showed continually increased aortic stiffness and no change in systolic BP (16). Although it is unknown whether systolic BP and aortic stiffness would continue to rise past week 12 in these WD-fed UM-HET3 mice, it is possible that systolic BP and aortic stiffness in female WD mice would start to elevate with a faster rate compared to male WD mice as metabolic function starts to deteriorate with aging in female WD mice (60). Thus, future studies are warranted in this area.

### Western diet may impair endothelium-dependent dilation by decreasing NO-mediated vasodilation

Carotid artery EDD was impaired in WD mice, demonstrated by blunted vasodilation to acetylcholine in WD compared to NC mice. Reductions in EDD appeared to be due to lower NO-mediated vasodilation in WD mice, indicating that EDD was reduced via decreases in NO bioavailability. We did not observe any blunted vasodilation to sodium nitroprusside. Thus, impaired EDD does not appear to be caused by dysfunction in vascular smooth muscle, as there was no impairment in EID. Moreover, the increase in systolic BP and aortic stiffness is linked with impaired EDD, but not EID (61, 62). Indeed, we observed strong inverse relationships between systolic BP or aortic stiffness and maximal vasodilation to acetylcholine in carotid artery. This relationship was not present between systolic BP or aortic stiffness and maximal vasodilation to sodium nitroprusside in carotid artery. Thus, it seems that the mechanism by which WD consumption induces elevation in systolic BP and aortic stiffening also plays a role in impaired EDD in carotid artery.

## Limitations

This study is not without limitations. We did not collect subcutaneous adipose tissue, therefore, was not able to determine whether sex-difference exits in subcutaneous adipose tissue mass as it has been shown that obese men typically have greater visceral adipose tissue and obese women have greater subcutaneous adipose tissue (63). We did not observe any differences in aortic lumen diameter, media CSA, or wall-to-lumen in WD mice. It should be noted that these measurements were derived from histological sections that were cut from unpressurized aortic rings. However, we also observed no differences in lumen diameter or wall thickness in pressurized carotid arteries that we used during *ex vivo* arterial function. While these data in pressurized carotid arteries support our histological findings in the aorta, further examination of the aorta structural characteristics when pressurized is warranted.

## Conclusion

In the present study, we observed a rapid increase in body mass in outbred, genetically diverse, male and female UM-HET3 mice fed WD. Interestingly, we observed WD resulted in metabolic dysfunction in male mice only, demonstrating a sex-specific manner by which WD impairs metabolic function in these mice. Although female WD mice did not develop metabolic dysfunction, they had greater visceral adiposity compared to male WD mice. Systolic BP and aortic stiffness also had a rapid increase in response to WD, which continued to increase through the end of the 12-week dietary intervention. Elevated systolic BP and aortic stiffness were strongly related to EDD, which was also impaired at the end of the intervention in male and female WD-fed mice. Although the precise physiological mechanism for these changes in response to WD is unclear, these data provide preliminary support for the use of UM-HET3 mice, an outbred, genetically diverse strain, as a mouse model for translational research on this topic. Future studies are warranted to elucidate the mechanism of WD on arterial aging and metabolic function in this mouse model, as they may provide important translational insight into sex-specific WD-induced metabolic and arterial dysfunction in industrialized human populations.

## Data availability statement

The raw data supporting the conclusions of this article will be made available by the authors, without undue reservation.

## Ethics statement

This animal study was reviewed and approved by Florida State University Animal Care and Use Committee.

## Author contributions

XZ, ZL, JBS, LS, CD, JB, and DM performed the experiments. DM prepared the figures. XZ and DM drafted the manuscript. All authors have analyzed the data, conception, design of research, interpreted results, edited, revised manuscript, and approved the final version of manuscript.
